# Thank you to reviewers 2025

**DOI:** 10.1093/ehjimp/qyag015

**Published:** 2026-02-25

We wish to thank the following reviewers for their contributions during 2025

**Top reviewers**

Nicolò De Biase

Valerio Di Fiore

Filippo Angelini

Federico Fortuni

Andrea Barison

Stefano Figliozzi

Alberto Aimo

Iacopo Fabiani

Ilir Sharka

Andrea Barbieri

**With thanks to all our 2025 reviewers**

Nicolò De Biase

Filippo Angelini

Federico Fortuni

Valerio Di Fiore

Andrea Barison

Stefano Figliozzi

Riccardo Liga

Alberto Aimo

Iacopo Fabiani

Ilir Sharka

Andrea Barbieri

Abdelrahman Elhakim

Alessia Gimelli

Valeria Pergola

Vincenzo Nuzzi

Nunzia Borrelli

Lucia La Mura

Konstantinos Katogiannis

Stefano Albani

Erika Hutt

Rebecca Perry

Vlatka Reskovic

Stefano Sforna

Rosaria Barracano

Sara Bombace

Vincenzo Positano

Francesco Angeli

Antonella Meloni

ASHIKA A

Bryan Abadie

Ana Almeida

Mahmoud Al Rifai

Ryo Horita

Georgios Georgiopoulos

Lavinia Del Punta

Lilit Baghdasaryan

Luca Franchin

Julia Grapsa

Ilaria Torre

Ignazio Gueli

Francesca Coraducci

Giovanni Novani

Matteo Mazzola

Andreas Giannopoulos

Lamia Ait Ali

Dan-Mihai Dorobantu

Chrysanthos Grigoratos

Giuseppe Ciliberti

Erik Andreas Berg

Faiza Al Kindi

Eirini Beneki

Rami Homsi

Tom Wang

Bo Xu

Yu Fu Ferrari Chen

Vinesh Appadurai

Sara Moscatelli

Tommaso Semino

Guglielmo Gioia

Pieter van der Bijl

Pietro Costantini

Zoran popovic

Raffaella Mistrulli

Sebastian Rosch

Tooba Akbari

Edoardo Zancanaro

Elias Andrade Cuellar

Stefania Angela Di Fusco

Dominik Benz

Anna Barton

Almudena Ortiz Garrido

Andrija Pavlovic

Benedetta Leonardi

Georgios Benetos

Roberto Baltodano-Arellano

Blanche Cupido

Matthias Unterhuber

Nils Johnson

Olivier Lairez

Ornela Velollari

Pankaj Garg

Pauline Gut

Per Arvidsson

Philip Haaf

Mariachiara Ciarlantini

Marta Cvijic

Michael Merhige

Michele Coceani

Maribel Gonzalez del Hoyo

Filippo Zilio

Fabrizio Ricci

Firas Al Badarin

Felipe Sanchez Tijmes

Luca Bergamaschi

Jolanda Sabatino

Joost Lumens

Jennifer von Stein

Konstantinos Vakalis

William Katz

Simrat Kaur

Masataka Ikeda

Imtiaz ul Hassan

Jennifer Erley

Jerome Garot

Fei Kang

Francesco Prattichizzo

Giulia De Zan

Elena Galli

Gianluca Pontone

Fausto Pizzino

Hatem Soliman-Aboumarie

Giancarlo Todiere

Mihaly Karolyi

Monica Sigovan

Nadjia Kachenoura

Nathaniel Murphy

Shahab Masoumi

Massimiliano Camilli

Marco Rolando

Margarita Brida

Krasimira Hristova

Jonathan Labin

Lukas Stolz

Magalie Viallon

Magdalena Gurzun

Pietro Scicchitano

Panagiota Mitropoulou

Maria Concetta Pastore

Hideharu Oka

Maximilian von Roeder

Stephanie Brunner

Blazej Michalski

Cornelis Van den Berg

Christoph Nienaber

Angelica Cersosimo

Aniela Popescu

Anne Schoeber

Antonella Cecchetto

Amer Barakat

Andrea Baggiano

Anna Kate Barton

Abdalla Abdelkader

Amanda Craine

Fatemah Aladwani

Navneet Kaur

Alexandros Kasiakogias

Alessandra Scatteia

Aju Pazhenkottil

Albert Roque

Alexander Gotschy

Waleed Alhumaid

Ambreen Ali

Francesco Bianco

Amalia Peix

Attila KovÃ¡cs

Aurelien Bustin

Aya Ghoul

Claudio Tinoco Mesquita

Cristian Herrera

Dominika SuchÃ¡

Danijela Trifunovic Zamaklar

David Marlevi

Milind Desai

Rosalba De Sarro

Emanuele Monda

Elizabeth Paratz

Erberto Carluccio

Erwan Donal

Knebel Fabian

Edoardo Conte

Edouard Gerbaud

Ankit Gupta

Ben Costello

Théo Richard

Theodor Lav

Thomas Kuestner

Thomas MD

Teresa Correia

Tomaz Podlesnikar

Rosa Sicari

Stefano Nistri

Sebastian Kelle

Tamanna Singh

Satoshi Nakamura

Sneha Vakamudi

Soumaya Sridi-Cheniti

Kimon Stamatelopoulos

Efstathios Pagourelias

Soufiane Ben Haddou

Simona Celi

Floran Sahiti

Rinchyenkhand Myagmardorj

Rob Eerdekens

Roberto Badagliacca

Rodolfo Lustosa

Rashid Alavi

Renee Bullock-Palmer

Zachary Rhinehart

Roberto Tarantini

Kyriakos Yiangou

Yasuhiro Tanabe

Valeria De Luca

Varun Pattisapu

Vladyslav Chubuchnyi

Victor de Villedon de Naide

Vinay Guduguntla

